# Branch retinal artery occlusion following radiation therapy to the head and neck: a case report

**DOI:** 10.1186/1471-2415-13-66

**Published:** 2013-11-01

**Authors:** Helen Jiang, Maxwell S Stem, Jerome I Finkelstein

**Affiliations:** 1 University of Michigan Medical School, Ann Arbor, USA; 2 Department of Ophthalmology and Visual Sciences, University of Michigan Medical School, Ann Arbor, USA

**Keywords:** Radiation, Head and neck, Atherosclerosis, Branch retinal artery occlusion (BRAO)

## Abstract

**Background:**

Previous studies have established that radiation to the head and neck leads to atherosclerosis and stenosis of the carotid artery and subsequent increased stroke risk, but the ophthalmic sequella following cervical irradiation is less well-defined.

**Case presentation:**

We present a single case of branch retinal artery occlusion (BRAO) in a 55 year-old Caucasian male seen at the University of Michigan in 2008 following unilateral head and neck radiation.

**Conclusion:**

This case demonstrates that patients receiving radiation to the head and neck may be at increased risk for developing a BRAO secondary to atherosclerotic changes of vessels adjacent to the radiation target. Given this risk, it may be reasonable to obtain carotid artery imaging in patients with a history of cervical radiation who present with sudden or transient visual field defects, even in the absence of other conventional risk factors for atherosclerosis.

## Background

Prior studies have demonstrated that radiation therapy to the head and neck leads to carotid artery stenosis [1]. Furthermore, head and neck radiation increases stroke risk [2]. However, the relationship between radiation-induced carotid artery damage and embolic ophthalmic events has been less well explored. We present a case of branch retinal artery occlusion (BRAO) in a patient without known cardiovascular disease following unilateral head and neck radiation.

### Case presentation

A 55-year-old Caucasian man with a history of squamous cell carcinoma of the left tonsil treated with neck dissection, chemotherapy, and radiation therapy eight years previously presented to a comprehensive ophthalmologist complaining of a gray spot in the upper right quadrant of his left eye that began four days ago. He denied any pain, flashes, or floaters. Past medical history was significant for hyperlipidemia, and family history was significant for coronary artery disease (CAD).

**Figure 1:**
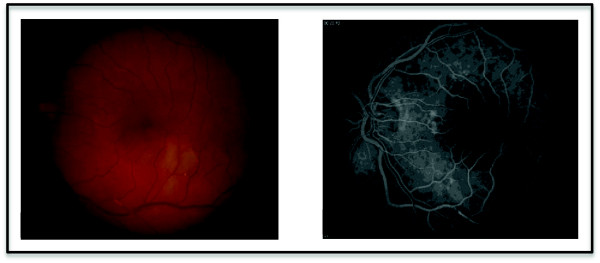
**The fundus photograph demonstrates multiple Hollenhurst plaques and an area of retinal edema in the left eye.** The fluoroscein angiogram highlights inferior vascular filling defects.

Examination of the left eye revealed a vision of 20/20, intraocular pressure of 16 mmHg, and a normal slit lamp exam (SLE). Dilated fundus exam (DFE) of the left eye demonstrated a region of retinal edema along the inferotemporal arcade extending just below the fovea. Multiple Hollenhorst plaques were noted throughout the same area, consistent with a diagnosis of BRAO. The fundus photograph and fluorescein angiogram from the day after presentation are shown in Figure 1. Examination of the right eye was normal.

The patient underwent carotid artery ultrasound, which revealed a fully patent right carotid but evidence of a heterogeneous plaque measuring 70-99% diameter stenosis in the left carotid. A transthoracic echocardiogram did not reveal any intracardiac sources of emboli. With his BRAO and significant ipsilateral carotid artery stenosis, the patient met clinical criteria for undergoing a carotid endarterectomy [3] and was referred to a vascular surgeon for this definitive treatment.

**Figure 2:**
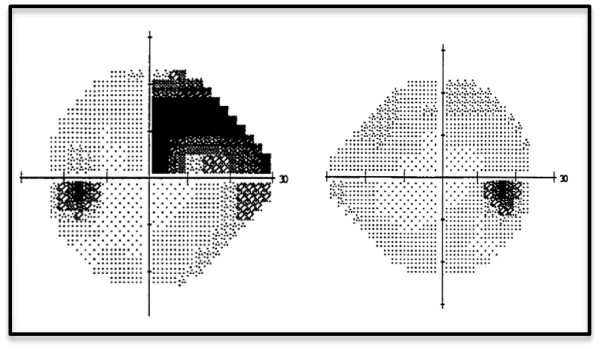
**Three months after presentation, the patient had a residual superonasal visual field defect in the left eye.**

Three months after the patient’s endarterectomy, he was noted to have 20/20 vision in the left eye with residual visual field defect in the superonasal quadrant of his left eye, corresponding to the original area of retinal nonperfusion (Figure 2).

## Conclusions

Radiation therapy promotes the development of atherosclerosis by damaging the vascular endothelium [1]. Radiation-induced vascular damage may occur through several potential mechanisms such as increased proteoglycan deposition and/or inflammatory cell infiltration [4].

Our patient presented with a BRAO of the left eye eight years after radiation therapy to the head and neck. The radiation-induced damage to the left carotid artery likely precipitated the BRAO. Arguably, the patient had several risk factors for conventional atherosclerosis including hyperlipidemia and a family history of CAD. However, the presence of significant stenosis in the carotid artery ipsilateral to the site of irradiation, the absence of atherosclerotic disease in the contralateral carotid, and the lack of atherosclerotic retinal vessels on DFE all support the hypothesis that radiation was the primary factor responsible for the patient’s left carotid artery stenosis. The ophthalmic literature contains one case of central retinal artery occlusion (CRAO) following neck irradiation [5]. However, that patient developed a CRAO contralateral to the site of irradiation and was found to have bilateral carotid stenosis on subsequent imaging. To the best of our knowledge, a case of unilateral radiation-induced atherosclerosis leading to BRAO has not been reported previously in the literature.

This case reiterates that patients who have received head and neck radiation may be at increased risk for the development of atherosclerosis in the arteries proximal to the radiation target. While this patient clearly had a BRAO based on fundoscopic exam, it is feasible that such patients could present with transient visual field defects or vision loss without any associated Hollenhorst plaques. Therefore, it may be prudent to obtain carotid artery imaging in patients with a history of head and neck radiation who present with sudden, transient, or prolonged visual field defects, even if the patients have few other risk factors for atherosclerosis or lack pathognomonic Hollenhorst plaques during fundoscopic examination.

### Consent

Written informed consent was obtained from the patient for publication of this Case report and any accompanying images. A copy of the written consent is available for review by the Editor of this journal.

## Competing interests

The authors declare that they have no competing interests.

## Authors’ contributions

MS and HJ performed a background search of the literature and drafted the manuscript. JF provided care for the patient, conceived of this case report, and helped to edit the manuscript. All authors read and approved the final manuscript.

## Pre-publication history

The pre-publication history for this paper can be accessed here:

http://www.biomedcentral.com/1471-2415/13/66/prepub
